# Recording of Influenza-Like Illness in UK Primary Care 1995-2013: Cohort Study

**DOI:** 10.1371/journal.pone.0138659

**Published:** 2015-09-21

**Authors:** Pia Hardelid, Greta Rait, Ruth Gilbert, Irene Petersen

**Affiliations:** 1 Population, Policy and Practice Programme, University College London Institute of Child Health, London, United Kingdom; 2 Research Department of Primary Care and Population Health, University College London, London, United Kingdom; 3 PRIMENT Clinical Trials Unit, Research Department of Primary Care and Population Health, University College London, London, United Kingdom; 4 Department of Clinical Epidemiology, Aarhus University, Aarhus, Denmark; New York City Department of Health and Mental Hygiene, UNITED STATES

## Abstract

**Background:**

There is a lack of recent studies examining recording of influenza-like illness (ILI) in primary care in the UK over time and according to population characteristics. Our aim was to determine time trends and socio-demographic patterns of ILI recorded consultations in primary care.

**Methods:**

We used The Health Improvement Network (THIN) UK primary care database and extracted data on all ILI consultations between 1995 and 2013. We estimated ILI recorded consultation rates per 100,000 person-weeks (pw) by age, gender, deprivation and winter season. Negative binomial regression models were used to examine time trends and the effect of socio-demographic characteristics. Trends in ILI recorded consultations were compared to trends in consultations with less specific symptoms (cough or fever) recorded.

**Results:**

The study involved 7,682,908 individuals in 542 general practices. The ILI consultation rate decreased from 32.5/100,000 pw (95% confidence interval (CI) 32.1, 32.9) in 1995–98 to 15.5/100,000 pw (95% CI 15.4, 15.7) by 2010–13. The decrease occurred prior to 2002/3, and rates have remained largely stable since then. Declines were evident in all age groups. In comparison, cough or fever consultation rates increased from 169.4/100,000 pw (95% CI 168.6, 170.3) in 1995–98 to 237.7/100,000 pw (95% CI 237.2, 238.2) in 2010–13. ILI consultation rates were highest among individuals aged 15–44 years, higher in women than men, and in individuals from deprived areas.

**Conclusion:**

There is substantial variation in ILI recorded consultations over time and by population socio-demographic characteristics, most likely reflecting changing recording behaviour by GPs. These results highlight the difficulties in using coded information from electronic primary care records to measure the severity of influenza epidemics across time and assess the relative burden of ILI in different population subgroups.

## Introduction

The influenza virus is a common cause of respiratory tract infections. Antigenic shift of the virus may lead to pandemics,[1] the most recent caused by influenza A/H1N1 in 2009.[2] Although the majority of infections are mild and self-limiting, influenza can cause severe complications leading to hospital admission or death.[3, 4] Nationally representative influenza surveillance systems are required by governments to implement timely prevention efforts to mitigate the effects of seasonal and pandemic influenza.

In the United Kingdom (UK) and many other countries, a cornerstone of clinical influenza surveillance is monitoring of patient consultations in primary care.[5, 6] These surveillance systems are based on extracts from primary care electronic records where a relevant diagnosis has been made. However, influenza is difficult to diagnose clinically, and only a small minority of suspected cases undergo confirmatory diagnostic tests in a primary care setting. Instead, general practitioners (GPs, primary care physicians) make decisions regarding diagnosis and management during a short consultation, based solely on presenting symptoms. Primary care surveillance is therefore based on a clinical diagnosis of influenza-like illness (ILI), rather than influenza itself. The symptoms included in the clinical definition of ILI include sudden onset of fever (above 38.5°C) and cough.[7, 8] However, ILI symptoms can be caused by a number of other viruses, including adenovirus, rhinovirus and human metapneumovirus.[9, 10] Conversely, persons with influenza may not receive a diagnosis of ILI when they consult primary care. A recent community study demonstrated that only 8% of a small sample of symptomatic individuals with confirmed influenza who consulted their GP had ILI recorded in their primary care record.[11]

Coding of ILI in primary care records may be driven by the incidence of influenza and other respiratory viruses, or unrelated factors. Such factors include clinician preference or recommendations regarding treatment, which may change over time, such as during pandemics.[12] ILI recording rates in primary care are also likely to be determined by demographic characteristics. For example, ILI consultation rates have been found to consistently vary by age group, with the highest consultation rates in young children compared to adults,[13, 14] and in women compared to men.[15] Studies of ILI recording in UK primary care are now dated.[15–17]. Our objectives were to determine long-term trends in recording of ILI in UK primary care, examine more recent recording patterns according to population socio-demographic characteristics, and variation in recording at GP practice level. We aimed to inform interpretation of data from surveillance systems for ILI based on electronic primary care records.

## Methods

### Data source

Around 98% of the UK population is registered with a GP.[18] The Health Improvement Network (THIN) is a database containing longitudinal primary care records from around 6% of the UK population, registered with GP practices that use VISION patient management software and have agreed to contribute data to THIN.[19] THIN contains data on prescriptions and diagnoses, together with additional demographic information. The registered THIN population is broadly representative of UK demography, and general practices contributing data to THIN are representative of UK general practices in terms of prescribing and consultation frequency.[20, 21]

Diagnoses and symptoms are entered in primary care electronic records using Read codes [22] by the GP, usually during patient consultations. Read codes are a coding system used to record clinical summary information. Two systematic reviews [23, 24] found that a high proportion of diagnoses recorded as Read codes in electronic medical records were confirmed using internal validation, or external validation using GP questionnaires or paper medical records. Prescriptions are entered using drug codes that map onto chapters of the British National Formulary.[25]

Severe outcomes of influenza are more common in persons of low socio-economic status,[26, 27] and consultations rates for lower respiratory tract infections are higher among individuals living in deprived areas.[28] We therefore examined the effect of socio-economic deprivation on ILI consultations, using quintiles of Townsend scores. The Townsend score is a small area based measure of multiple deprivation based on data from the 2001 Census. It takes into account property and car ownership, overcrowding and unemployment in the resident population of a small area consisting of approximately 150 households. In THIN, the Townsend score is linked to a patient through their postcode of residence by the data providers; however the actual postcodes and lower level geographies are not made available to researchers due to concerns regarding anonymity.

### Study population and period

We included all individuals registered in a contributing THIN practice from birth up to 99 years inclusive, who were registered for at least seven days at any point during the study period, 2^nd^ October 1995 to 19^th^ May 2013. Patient electronic records were included from the date at which practices met quality criteria regarding data entry.[29, 30] We examined consultations occurring in the winter seasons 1995/1996 to 2012/2013, where a winter season is defined as Monday week 40 (beginning of October) in year *x* to Sunday in week 20 (middle of May) in year *x+1*.[31]

### Definition of influenza-like illness (ILI)

We used established methods based on word searches of the Read code dictionary,[32] to create a code list for ILI (S1 Table), including codes for influenza-like illness, influenza and positive swab results for influenza. These codes were chosen as they indicate clinicians’ willingness to record a specific diagnosis of ILI or influenza infection (even if only suspected). We also included prescriptions of neuraminidase inhibitors (NIs, oseltamivir or zanamivir) as indicators of ILI.

We compared trends in recording of specific ILI codes to trends in recording of cough or fever (S1 Table), as an indicator of recording of non-specific symptoms of respiratory tract infections. We chose fever or cough as these two symptoms are recommended for community influenza surveillance by the World Health Organisation,[8] although only a minority of cough or fever episodes would be expected to be caused by influenza.[11] Since it is rare for GPs to record two different diagnoses during a consultation, we did not examine recording of cough or fever during the same consultation. We included only the first ILI record per person within each season as infection with two different influenza strains during a season is rare,[33] and only a small minority (around 1%) of persons consulting with ILI consulted twice or more during a season. Likewise, we only included the first cough or fever consultation per person per season in order to carry out similar comparisons over time between specific and non-specific diagnoses.

### Statistical methods

#### Time trends in ILI recorded consultations, 1995–2013

We calculated consultation rates for ILI and cough or fever symptoms per 100,000 person-weeks with 95% confidence intervals (CI), according to gender, age group (coded into standard groups for influenza, [34, 35] see S2 Table), quintiles of Townsend score and winter season. We validated the ILI consultation rate in THIN in 2010 against the ILI consultation rate from a separate primary care surveillance scheme in England, run by the Royal College of General Practitioners (RCGP).[36]

We calculated the percentage change in ILI and cough or fever consultation rates between the first three seasons (1995/96, 1996/97 and 1997/98) and the last three seasons (2010/11, 2011/12 and 2012/13) of the study period.

Details of model fitting are fully described in S1 text. Briefly, we fitted negative binomial regression models with number of ILI consultations in a winter season as the outcome variable, ordered winter season (eg. 1995/96 = 1, 1996/97 = 2) as the predictor variable (using restricted cubic splines) and person-time at risk as the offset. We varied the number of internal knots of the splines from one to five to obtain the best fit to the data. The knots were placed at equally spaced percentiles of the distribution of ordered winter season, as suggested by Harrell.[37] Age group, gender, Townsend quintile and a pandemic season indicator were added as covariates. Interaction terms between ordered winter season: age group, and the pandemic indicator: age group were included to examine whether time trends were significantly different according to age group. We used minimisation of Akaike’s Information Criterion (AIC) to determine whether the inclusion of a particular variable significantly improved the fit of the model.

#### Recent recoding of ILI according to socio-demographic characteristics, 2010–2013

To examine recent ILI recording according to socio-demographic variables, we fitted negative binomial regression models with number of ILI recorded consultations as the outcome variable, age group, gender, winter season, Townsend quintile and a gender: age group interaction term as predictor variables and person-time at risk as the offset, to data from the last three seasons of the study period. The AIC was used to determine whether a particular variable significantly improved the fit of the model. We calculated the proportion of the registered population at the start of each season who had at least one consultation for ILI during that season, by age group, gender and Townsend quintile, with 95% confidence intervals.

#### Practice-level variation in ILI recorded consultations, 2010–2013

We predicted the number of consultations with an ILI recorded by practice, based on the model with age, gender, winter season and Townsend quintile, and divided the observed by the predicted number of ILI consultations to calculate standardised consultation ratios (SCRs). We used funnel plots,[38] with 95% overdispersion-adjusted control limits to compare the SCRs against an SCR of 1, which would be expected if observed and expected consultation rates were equal.

All statistical analyses were carried out using Stata version 13.[39]

### Ethics

All data were anonymised. THIN data collection has been approved by the South East NHS Multicentre Research Ethics Committee. The analyses for this study were approved by the Scientific Review Committee of the data providers (CSD Medical Research, now IMS Health), study reference number SRC 14–004.

## Results

The study included 7,682,908 individuals from 542 general practices, who had 287,320 first episodes of ILI during the 18 winter seasons in the study period. S2 Table shows the distribution of socio-demographic variables in the study population. The overall ILI consultation rate during winter seasons was 17.1/100,000 person-weeks (pw; 95% CI 17.0, 17.1 per 100,000 pw). In 2010, the overall rate of ILI was 12.2/100,000 pw in THIN and 11.8/100,000 pw in the RCGP network.

### Time trends in ILI recorded consultations, 1995–2013

**Fig 1:**
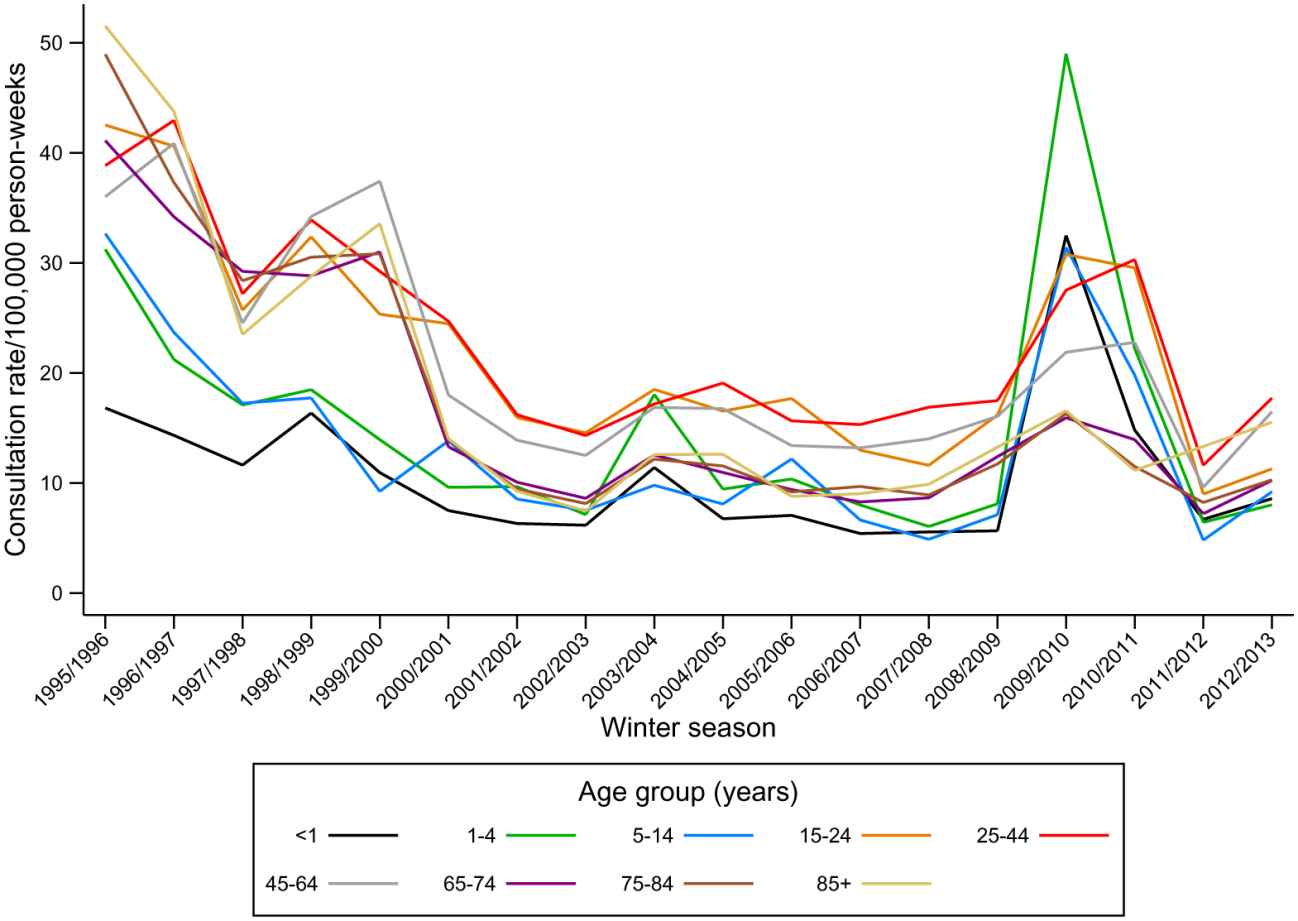
Consultation rates for ILI (per 100,000 person-weeks) by age group and winter season, 1995–2013.

**Fig 2:**
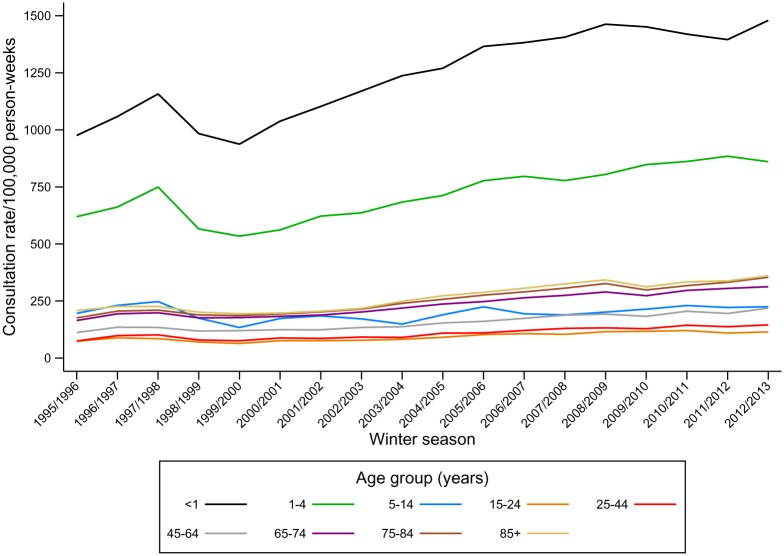
Consultation rates for cough or fever symptoms (per 100,000 person-weeks) by age group and winter season, 1995–2013*. *Note difference in scale on y-axis cf. Fig 1.

ILI consultation rates decreased by 52% during the study period (Fig 1); from 32.5/100,000 pw in the first three seasons, 1995/96, 1996/97 and 1997/98, (95% CI 32.1, 32.9) to 15.5/100,000 pw (95% CI 15.4, 15.7) in the last three seasons (2010/11, 2011/12 and 2012/13). This decline occurred prior to 2002/3 (S3 Table). After this date ILI consultation rates remained largely stable, apart from year-on-year variations and the peak in consultations during the 2009/10 pandemic (Fig 1). In contrast, consultation rates for non-specific cough or fever symptoms increased by 40% from 169.4/100,000 pw (95% CI 168.6, 170.3) to 237.7/100,000 pw (95% CI 237.2, 238.2) between the first three and the last three seasons (Fig 2, S3 Table).

A cubic spline with four internal knots at 2002/03, 2004/05, 2007/08 and 2009/10 best explained the time trend in ILI consultations, based on minimisation of the AIC. Including the age group: season, and age group: pandemic indicator interaction terms significantly improved model fit, implying that the observed trends over time in ILI recorded consultations vary significantly by age group. S1 Fig shows the fit of the final models. An overall decline prior to 2002/3 in ILI consultations was observed in all age groups, but the largest declines were observed in individuals aged 65 years or above. Children aged less than 15 years experienced the highest peak in consultations during the 2009–2010 pandemic.

### Recent recoding of ILI according to socio-demographic characteristics, 2010–2013

Overall, ILI consultation rates were higher among women than men, but this difference varied by age group (an age group: gender interaction term significantly improved model fit based on minimisation of AIC). Consultation rates were higher in women than in men among individuals aged between 15 and 74 years. Only small gender differences in consultation rates were observed in children aged less than 15 years and persons aged over 75 years (Table 1). Individuals living in more deprived areas had higher consultation rates (incidence rate ratio comparing highest versus lowest Townsend deprivation quintile 1.25 (1.14, 1.37).

**Table 1:** Observed consultation rates and adjusted incidence rate ratios by age group, gender, winter season and Townsend quintile, 2010–2013*.

Variable	Consultation rates/100,000 pw (95% CI)	Adjusted incidence rate ratios^†^ (95% CI)
**Townsend quintile**		
1^st^ (least deprived)	14.1 (13.9, 14.4)	1 (baseline)
2^nd^	14.6 (14.3, 14.8)	1.04 (0.99, 1.09)
3^rd^	15.8 (15.5, 16.1)	1.13 (1.05, 1.20)
4^th^	16.6 (16.3, 16.9)	1.18 (1.09, 1.28)
5^th^ (most deprived)	17.8 (17.4, 18.2)	1.25 (1.14, 1.37)
**Season**		
2010/11	23.5 (23.2, 23.8)	1 (baseline)
2011/12	9.2 (9.0, 9.3)	0.39 (0.37, 0.41)
2012/13	13.9 (13.7, 14.1)	0.59 (0.56, 0.62)
	**Men**	
**Age group (years)**		
<1 year	11 (9.6, 12.5)	0.66 (0.49, 0.88)
1–4 years	12.3 (11.6, 13.1)	0.73 (0.65, 0.83)
5–14 years	11.2 (10.8, 11.7)	0.66 (0.61, 0.72)
15–24 years	13.5 (13.0, 14.0)	0.80 (0.76, 0.84)
25–44 years	16.7 (16.3, 17.0)	1 (baseline)
45–64 years	13.5 (13.1, 13.8)	0.84 (0.80, 0.87)
65–74 years	9.1 (8.6, 9.6)	0.57 (0.53, 0.60)
75–84 years	9.5 (8.8, 10.1)	0.59 (0.53, 0.65)
85–99 years	12.5 (11.2, 13.9)	0.77 (0.65, 0.92)
	**Women**	
**Age group**		
<1 year	9.1 (7.8, 10.6)	0.39 (0.30, 0.51)
1–4 years	12.2 (11.4, 12.9)	0.52 (0.46, 0.59)
5–14 years	11.4 (11.0, 11.9)	0.49 (0.45, 0.52)
15–24 years	19.8 (19.2, 20.4)	0.85 (0.82, 0.88)
25–44 years	23.3 (22.8, 23.7)	1 (baseline)
45–64 years	19.2 (18.8, 19.5)	0.84 (0.81, 0.87)
65–74 years	11.7 (11.2, 12.2)	0.53 (0.50, 0.56)
75–84 years	10.4 (9.9, 11.0)	0.47 (0.43, 0.51)
85–99 years	13.7 (12.7, 14.7)	0.61 (0.47, 0.79)

*Model included 507 practices with data from the seasons 2010/11-2012/13.

^**†**^Model included linear predictors for age group, gender, Townsend score, season and an age group:gender interaction term.

Only 0.5% of the registered population had a record of at least one ILI consultation during the last three winters (combined) of the study period. Even in the age, gender and deprivation groups with the highest consultation rates, the proportion with one or more ILI consultations during a winter season did not exceed 1% (S2 Fig).

### Practice-level variation in ILI recorded consultations, 2010–2013

**Fig 3:**
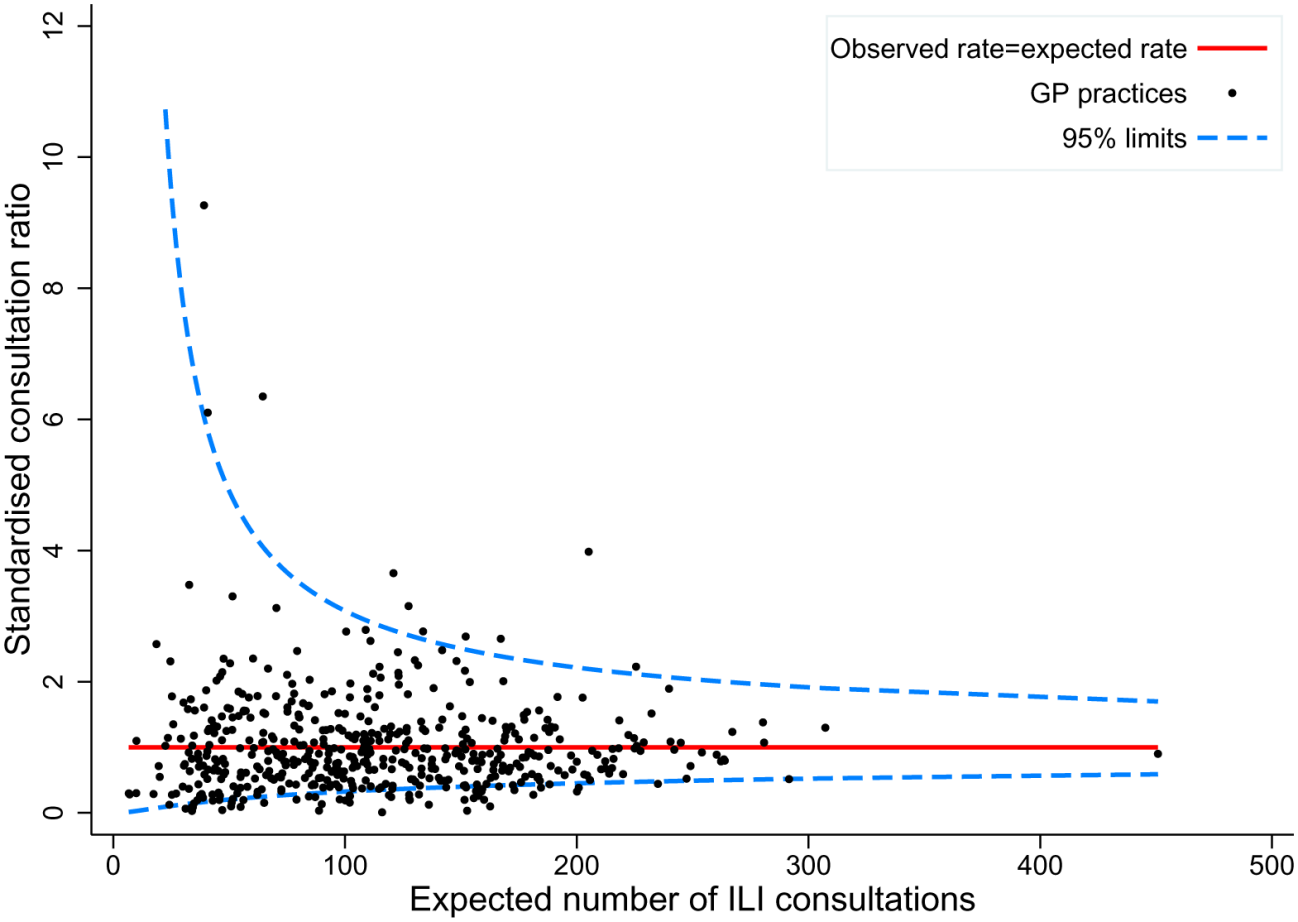
Funnel plot of standardised ILI consultation ratios (SCRs) by practice, 2010/11-2012/13*. *SCRs are adjusted for age group, sex, Townsend quintile and winter season.

Practice-level ILI consultation rates varied between 0.1/100,000 pw to 154.3/100,000 pw. Of 507 practices contributing data in the three year period, 60 (11.8%) had SCRs which fell outside the 95% control limits of the funnel plot, whereas only 25 practices would be expected to by chance (Fig 3). Ten of 60 of the outlier practices had rates above the 95% control limit (17%) and 50 below.

## Discussion

ILI consultation rates in UK primary care declined during the late 1990s and early 2000s, leading to a halving of consultation rates during the study period. In contrast, recording of less specific symptoms of respiratory tract infections increased by 40% during the same period. The decline in ILI consultation rates was observed across all age groups. Women aged between 15 and 75 years were more likely than men to have an ILI consultation recorded, and older children and adults had the highest ILI consultation rates. We also identified substantial between-practice variation in ILI consultation rates.

### Strength and limitations

This was a large study of over seven million individuals, demographically representative of the UK population, across 18 winters. The large study size and long period of data collection allowed examination of consultation rates according to population subgroups, between practices and over time.

THIN is a database collected for clinical management, not research. This leads to some limitations. THIN only covers individuals who present to their GP. However a substantial proportion of people with ILI do not consult their GP,[11] therefore the ILI burden seen and recorded in primary care is an underestimate of the true population burden. We used the Townsend score, a small-area level indicator of deprivation, and not an individual level measure of a person’s socio-economic circumstances. This could result in misclassification of an individual’s deprivation status, in turn leading the observed association between deprivation level and ILI consultation rates to be underestimated.

### Interpretation

There are few studies of temporal trends in ILI consultations in the UK, and no repeated community or seroprevalence studies of influenza covering the whole study period. Declining ILI consultation rates have been observed in a sentinel surveillance network in England between 1966 and 2006, including during the late 1990s, but the magnitude of the decline was not reported, with no age-specific analyses.[16] Contemporary surveillance reports noted a reduction in influenza activity in England and Wales in the early 2000s compared to the late 1990s based on both primary care surveillance and laboratory data. [40] Declines in ILI consultation rates during the 1990s and 2000s have also been observed in primary care or outpatient settings in the Netherlands,[14] New Zealand [13] and Taiwan.[41]

Since not only influenza causes ILI, factors unrelated to influenza activity are also likely to contribute to the observed decline in ILI consultations. One likely explanation for the reduction in ILI consultation rates is changes coding by GPs, in favour of less specific codes. This is supported by our finding of a concurrent 40% increase in coding of cough or fever symptoms, indicating that it is not the propensity to consult GPs with common symptoms of respiratory tract infections which are driving the trends. Universal influenza vaccination for individuals aged 65 years and over was introduced in the UK in 2000.[42] It is possible that the introduction of influenza vaccine could lead GPs to be more reluctant to use codes specifically mentioning influenza and (such as the codes for ‘influenza-like’ or ‘flu-like’ illness) in vaccinated individuals. However, since declines in ILI consultation rates were observed in all age groups, with the main decline observed during the late 1990s, this is an unlikely explanation of our findings.

An increase in the use of non-specific codes in primary care databases has also been observed for certain chronic conditions.[43] In addition, UK public health information campaigns since the late 1990s encouraged self-management of symptoms of respiratory tract infections in order to reduce antibiotic prescribing.[44] An increase in the use of codes for less specific respiratory tract infections and ill-defined symptoms in children have been linked to GPs’ decision making regarding whether to describe antibiotics for symptoms of likely viral origin.[45]

We observed large between-practice differences in ILI coded consultation rates, most likely reflecting differences in GP recording preferences in the absence of standard diagnostic criteria for ILI. Local variation in the propensity to consult primary care or variation in local population infection rates may also contribute to the observed variation. More detailed data on practice locations and their proximity are required to determine the potential role of local outbreaks of respiratory infection in driving these differences.

Only a very minor proportion of registered individuals have an ILI recorded in their GP records during a winter season. This is to be expected from a recent community study, where even among confirmed, symptomatic influenza cases, only 21% consult their GP.[11] Apart from the 2009/10 pandemic, the highest ILI recorded consultation rates were observed in adults. This finding was also reported by Meier et al for UK primary care [17], but in New Zealand [13] and the Netherlands [14] ILI consultation rates were highest in children. Lower ILI consultation rates in children than adults are in contrast to community and seroepidemiological studies of influenza, in which children tend to have the highest infection rates.[11, 46, 47] Similarly, studies estimating the impact of influenza on hospital admissions in a number of countries have found the highest impact among young children as well as older adults.[48, 49] This discrepancy reflects differences in GPs’ willingness to assign specific ILI codes in young children with respiratory symptoms in whom competing diagnoses are harder to rule out. In contrast clear diagnostic guidelines had been issued during the 2009/10 pandemic; this is also when ILI consultation rates were highest among children. The reluctance to code ILI in children could lead to an underestimate of the burden of ILI, and therefore also the severity of influenza epidemics in children. A qualitative or questionnaire study examining why GPs decide to enter a specific ILI code in the absence of standard diagnostic criteria is required to understand these differences by age group.

Women were more likely to have an ILI diagnosis recorded than men among older children and adults. A higher ILI consultation rate in women than men has also been reported in a previous study.[15] In the UK, adult women are more likely to present to primary care than men. [50] However, women were also more likely to report ILI symptoms in an internet based community survey, even after adjusting for contact with children, suggesting other contributing factors including sex-based immunological differences. [51]

Rates of recorded ILI in primary care have declined in all age groups in the UK since 1995, whilst recording of less specific symptoms, cough or fever, has increased. The age pattern of ILI recorded in primary care differs from the age pattern of ILI and influenza observed in community settings in the UK. Our results suggest that differences in GP coding habits may explain these results. Policy makers and researchers need to be aware of the importance of GP coding behaviour. Observed differences in ILI consultations rates in electronic primary care records over time and between groups may not reflect true variability in the severity of influenza epidemics in the population.

## Supporting Information

S1 Fig Observed and expected ILI consultation rates/100,000 pw from best fitting model, by age group. Prediction is for men in the least deprived Townsend quintile.
(TIF)

S2 Fig Proportion of the registered population with at least one consultation record of ILI, 2010/11-2012/13, by age group, gender and Townsend quintile (least vs. most deprived). (TIF)

S1 Table Read code lists for influenza-like illness (ILI) and cough or fever symptoms. (DOCX)

S2 Table Distribution of key socio-demographic variables in the study sample. (DOCX)

S3 Table Observed number of events and consultation rates by age group, country, gender season and Townsend score in winter seasons 1995–2013. (DOCX)

S1 Text Supplementary methods. (DOCX)
