# Supplementary figures and images for: Recording of Influenza-Like Illness in UK Primary Care 1995-2013: Cohort Study
Source: PLoS One. 2015 Sep 21;10(9):e0138659. doi: 10.1371/journal.pone.0138659 (PMC4577110; doi:10.1371/journal.pone.0138659)

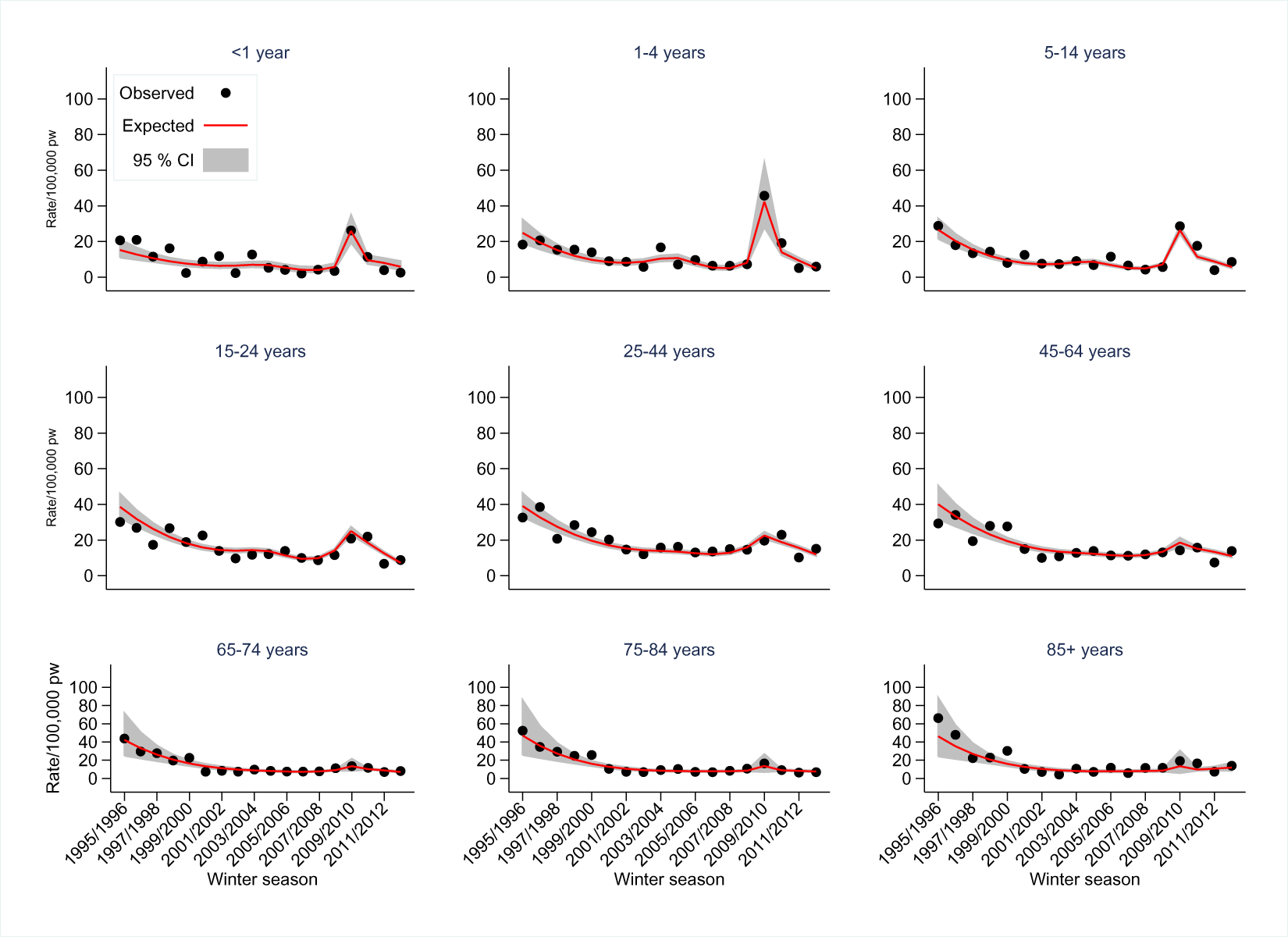

Supplement: S1 Fig — Prediction is for men in the least deprived Townsend quintile. (TIF) [file pone.0138659.s001.tif]

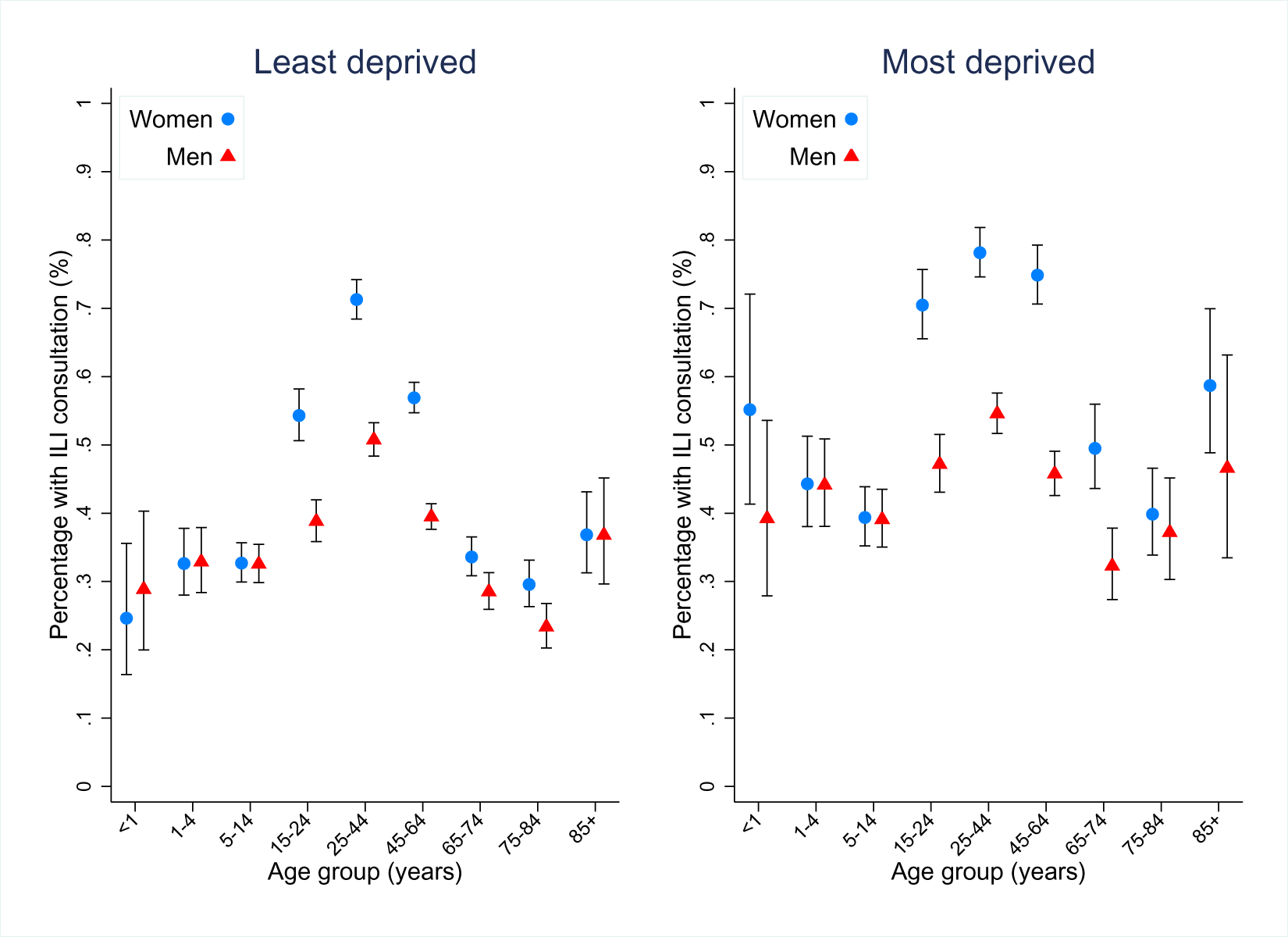

Supplement: S2 Fig — (TIF) [file pone.0138659.s002.tif]
