# Supplementary material for: Recording of Influenza-Like Illness in UK Primary Care 1995-2013: Cohort Study
Source: PLoS One. 2015 Sep 21;10(9):e0138659. doi: 10.1371/journal.pone.0138659 (PMC4577110; doi:10.1371/journal.pone.0138659)
Supplement: S1 Table — (DOCX) [file pone.0138659.s003.docx]

**S1 Table. Read code lists for influenza-like illness (ILI) and cough or fever symptoms**

| **Influenza-like illness (ILI)** | |
| --- | --- |
| **Read code** | **Description** |
| 16L..00 | Influenza-like symptoms |
| 1J72.00 | Suspected influenza A virus subtype H1N1 infection |
| 1J72.11 | Suspected swine influenza |
| 1W0..00 | Possible influenza A virus H1N1 subtype |
| 43dF.00 | Influenza A antibody level |
| 43dG.00 | Influenza B antibody level |
| 43k2.00 | Influenza A antigen level |
| 43k3.00 | Influenza B antigen level |
| 43w6.00 | Influenza A nucleic acid detection |
| 43wD.00 | Influenza B nucleic acid detection |
| 4J3L.00 | Influenza A virus H1N1 subtype detected |
| 4J3M.00 | Influenza A virus H1N1 subtype not detected |
| 4JDb.00 | Influenza (A&B) serology |
| 4JDe.00 | Influenza A virus subtype H1N1 serology |
| 4JU0.00 | Influenza H1 virus detected |
| 4JU1.00 | Influenza H2 virus detected |
| 4JU2.00 | Influenza H3 virus detected |
| 4JU3.00 | Influenza H5 virus detected |
| 4JU4.00 | Influenza A virus, other or untyped strain detected |
| 4JU5.00 | Influenza B virus detected |
| 4JU6.00 | Oseltamivir resistant virus detected |
| 4JU7.00 | Zanamivir resistant virus detected |
| 4JU8.00 | Amantadine resistant virus detected |
| 9G5..00 | Local flu response centre notified |
| 9N31000 | Telephne consultatn suspected influenza A virus subtype |
| 9N31011 | Telephone consultation for suspected swine flu |
| F030800 | Encephalitis due to influenza-specific virus not identified |
| F030A00 | Encephalitis due to influenza-virus identified |
| G520300 | Acute myocarditis - influenzal |
| H2...00 | Pneumonia and influenza |
| H27..00 | Influenza |
| H270.00 | Influenza with pneumonia |
| H270.11 | Chest infection - influenza with pneumonia |
| H270000 | Influenza with bronchopneumonia |
| H270100 | Influenza with pneumonia, influenza virus identified |
| H270z00 | Influenza with pneumonia NOS |
| H271.00 | Influenza with other respiratory manifestation |
| H271000 | Influenza with laryngitis |
| H271100 | Influenza with pharyngitis |
| H271z00 | Influenza with respiratory manifestations NOS |
| H27y.00 | Influenza with other manifestations |
| H27y000 | Influenza with encephalopathy |
| H27y100 | Influenza with gastrointestinal tract involvement |
| H27yz00 | Influenza with other manifestations NOS |
| H27z.00 | Influenza NOS |
| H27z.11 | Flu like illness |
| H27z.12 | Influenza like illness |
| H29..00 | Avian influenza |
| H2A..00 | Influenza due to Influenza A virus subtype H1N1 |
| H2A..11 | Influenza A (H1N1) swine flu |
| H2y..00 | Other specified pneumonia or influenza |
| H2z..00 | Pneumonia or influenza NOS |
| Hyu0400 | [X]Flu+oth respiratory manifestations,'flu virus identified |
| Hyu0500 | [X]Influenza+other manifestations,influenza virus identified |
| Hyu0600 | [X]Influenza+oth respiratory manifestatns,virus not identified |
| Hyu0700 | [X]Influenza+other manifestations, virus not identified |
|  |  |
| **Cough or fever symptoms** | |
| **Read code** | **Description** |
| 14OS.00 | Green traff light - low risk serious illness |
| 14OT.00 | Amber traff light - intermediate risk serious illness |
| 14OV.00 | Red traffic light - high risk of serious illness |
| 165..00 | Temperature symptoms |
| 165..11 | Fever symptoms |
| 165..12 | Pyrexia symptoms |
| 1652.00 | Feels hot/feverish |
| 1653.00 | Fever with sweating |
| 1654.00 | Having rigors |
| 1654.11 | Rigor - symptom |
| 1655.00 | C/O shivering |
| 1656.00 | Feverish cold |
| 165Z.00 | Temperature symptom NOS |
| 171..00 | Cough |
| 171..11 | C/O - cough |
| 171..12 | Sputum - symptom |
| 1712.00 | Dry cough |
| 1713.00 | Productive cough -clear sputum |
| 1714.00 | Productive cough -green sputum |
| 1715.00 | Productive cough-yellow sputum |
| 1716.00 | Productive cough NOS |
| 1716.11 | Coughing up phlegm |
| 1719.00 | Chesty cough |
| 1719.11 | Bronchial cough |
| 171F.00 | Cough with fever |
| 171Z.00 | Cough symptom NOS |
| 2E...00 | Examination of fever |
| 2E...11 | O/E - fever |
| 2E1..00 | O/E - fever - general |
| 2E13.11 | O/E - pyrexia - ? cause |
| 2E2..00 | O/E - method fever registered |
| 2E3..00 | O/E - level of fever |
| 2E34.00 | O/E - temperature elevated |
| 2E35.00 | O/E - hyperpyrexia-> 40.5 oCEL |
| 2E3Z.00 | O/E - level of fever NOS |
| 2E4..00 | O/E - character of fever |
| 2E4..11 | O/E - temperature character |
| 2E41.00 | O/E - fever - acute rise |
| 2E42.00 | O/E - fever - gradual rise |
| 2E43.00 | O/E - fever - continuous |
| 2E44.00 | O/E - fever - remittent |
| 2E45.00 | O/E - fever - intermittent |
| 2E47.00 | O/E - fever - irregular |
| 2E48.00 | O/E - fever - fast fall-crisis |
| 2E49.00 | O/E - fever-gradual fall-lysis |
| 2E4Z.00 | O/E - fever character NOS |
| A782.00 | Sweating fever |
| H00..13 | Febrile cold |
| H00..15 | Pyrexial cold |
| R006000 | [D]Chills with fever |
| R006100 | [D]Hyperpyrexia NOS |
| R006200 | [D]Fever NOS |
| R006300 | [D]Persistent fever |
