# Supplementary material for: Recording of Influenza-Like Illness in UK Primary Care 1995-2013: Cohort Study
Source: PLoS One. 2015 Sep 21;10(9):e0138659. doi: 10.1371/journal.pone.0138659 (PMC4577110; doi:10.1371/journal.pone.0138659)
Supplement: S2 Table — (DOCX) [file pone.0138659.s004.docx]

**S2 Table Distribution of key socio-demographic variables in the study sample**

| **Variable** | **Total^*^ number of patients (%)** |
| --- | --- |
| **Age at start of follow-up**  <1 year  1-4 years  5-14 years  15-24 years  25-44 years  45-64 years  65-74 years  75-84 years  85-99 years | 650320 (8.5)  391220 (5.1)  728584 (9.5)  1132204 (14.7)  2461580 (32.0)  1420693 (18.5)  449277 (5.8)  308386 (4.0)  140644 (1.8) |
| **Townsend quintile^†^**  1^st^ (least deprived)  2^nd^  3^rd^  4^th^  5^th^ (most deprived) | 1792862 (23.3)  1575902 (20.5)  1631464 (21.2)  1558327 (20.3)  1124353 (14.6) |
| **Gender**  Female  Male | 3945283 (51.4)  3737625 (48.6) |

^*^Total number of patients in study sample: 7,682,908

^†^253,867 patients had a missing Townsend score (3.3% of total); they were excluded prior to analyses.
