# Supplementary material for: Recording of Influenza-Like Illness in UK Primary Care 1995-2013: Cohort Study
Source: PLoS One. 2015 Sep 21;10(9):e0138659. doi: 10.1371/journal.pone.0138659 (PMC4577110; doi:10.1371/journal.pone.0138659)
Supplement: S3 Table — (DOCX) [file pone.0138659.s005.docx]

**S3 Table Observed number of events and consultation rates by age group, country, gender, season and Townsend score in winter seasons 1995-2013**

|  | **Consultation rates (events/100,000 person-weeks)** | | | |
| --- | --- | --- | --- | --- |
| **Variable** | **Influenza-like illness (ILI)** | | **Cough or fever symptoms** | |
|  | Number of consultations | Consultation rate/100,000pw  (95% CI) | Number of consultations | Consultation rate/100,000pw  (95% CI) |
| **Age group**  <1 year  1-4 years  5-14 years  15-24 years  25-44 years  45-64 years  65-74 years  75-84 years  85-99 years | 1770  10887  22548  35838  98679  79242  20078  13177  5101 | 10.3 (9.8, 10.8)  14.2 (14.0, 14.5)  11.8 (11.7, 12.0)  18.9 (18.7, 19.1)  20.4 (20.3, 20.6)  17.9 (17.8, 18.1)  13.3 (13.2, 13.5)  13.5 (13.2, 13.7)  14.3 (14.0, 14.7) | 189652  497367  364821  186005  541851  719239  361284  255099  97569 | 1302.1 (1296.2, 1307.9)  747.6 (745.5, 749.7)  197.9 (197.2, 198.5)  99.5 (99.0, 99.9)  114.1 (113.8, 114.4)  167.0 (166.7, 167.4)  249.9 (249.1, 250.7)  272.2 (271.2, 273.3)  286.9 (285.1, 288.7) |
| **Townsend quintile**  1^st^ (least deprived)  2^nd^  3^rd^  4^th^  5^th^ (most deprived) | 68878  57823  60675  57277  42667 | 15.5 (15.3, 15.6)  15.7 (15.6, 15.8)  17.4 (17.3, 17.6)  18.5 (18.4, 18.7)  20.2 (20.0, 20.4) | 783332  658314  674784  633639  462818 | 180.9 (180.5, 181.3)  183.9 (183.5, 184.4)  200.2 (199.7, 200.6)  212.2 (211.6, 212.7)  227.7 (227.0, 228.3) |
| **Gender**  Male  Female | 126191  161129 | 15.1 (15.0, 15.2)  19.0 (18.9, 19.1) | 1434130  1778757 | 176.7 (176.4, 177.0)  217.3 (217.0, 217.6) |
| **Winter season**  1995/96  1996/97  1997/98  1998/99  1999/00  2000/01  2001/02  2002/03  2003/04  2004/05  2005/06  2006/07  2007/08  2008/09  2009/10  2010/11  2011/12  2012/13 | 8136  10989  8777  13722  16957  14029  11748  11771  17052  18204  16400  14929  15478  18473  33505  29109  11380  16661 | 38.3 (37.4, 39.1)  37.6 (36.9, 38.3)  24.8 (24.3, 25.4)  30.4 (29.9, 30.9)  28.1 (27.7, 28.6)  19.0 (18.7, 19.3)  13.2 (13.0, 13.5)  11.7 (11.5, 11.9)  15.6 (15.4, 15.8)  15.1 (14.9, 15.3)  13.5 (13.3, 13.7)  12.0 (11.8, 12.2)  12.2 (12.0, 12.4)  14.4 (14.2, 14.6)  25.9 (25.7, 26.2)  23.5 (23.2, 23.8)  9.2 (9.0, 9.3)  13.9 (13.7, 14.1) | 30808  49498  61790  64948  82049  109555  134736  158035  175590  216403  232332  247149  260275  275525  273035  281974  278082  281103 | 147.4 (145.8, 149.1)  173.2 (171.7, 174.7)  179.7 (178.3, 181.1)  146.8 (145.7, 147.9)  138.7 (137.8, 139.7)  151.6 (150.7, 152.5)  155.6 (154.8, 156.5)  161.3 (160.5, 162.1)  164.8 (164.0, 165.6)  184.9 (184.1, 185.7)  197.9 (197.1, 198.7)  205.5 (204.7, 206.3)  213.1 (212.3, 213.9)  222.5 (221.7, 223.3)  218.8 (217.9, 219.6)  236.3 (235.4, 237.2)  232.5 (231.6, 233.3)  244.5 (243.6, 245.4) |
