# Supplementary material for: Recording of Influenza-Like Illness in UK Primary Care 1995-2013: Cohort Study
Source: PLoS One. 2015 Sep 21;10(9):e0138659. doi: 10.1371/journal.pone.0138659 (PMC4577110; doi:10.1371/journal.pone.0138659)
Supplement: S1 Text — (DOCX) [file pone.0138659.s006.docx]

**S1 Text. Supplementary methods for ‘Influenza-like illness consultations in UK primary care 1995-2013: cohort study’**

**Model fitting and selection for time trends in ILI consultations 1995-2013**

We fitted negative binomial regression models with count of the number of ILI consultations as the outcome variable, ordered winter season (eg. 1995/96=1, 1996/97=2) as the predictor variable and person-time at risk as the offset. We first included ordered winter season as a linear term to examine whether time trends were significant. We then included ordered winter season as a restricted cubic spline, varying the number of internal knots from one to five to obtain the best fit to the data. The knots were placed at equally spaced percentiles of the distribution of ordered winter season, as suggested by Harrell.[[1](#_ENREF_1)] Age group, gender and Townsend quintiles were then added as covariates. We expected a peak in consultations during the winter of 2009/10 when pandemic influenza A/H1N1 was circulating. We therefore also included an indicator variable for the pandemic season (which was =1 in 2009/10 and =0 otherwise). Based on preliminary analyses we examined interactions between ordered winter season : age group, and pandemic indicator : age group , to examine whether the observed trends in ILI recording were significantly different by age group.

For all regression models, we used minimisation of the Akaike Information Criterion (AIC) as the criterion to select the model of best fit. Robust estimates of variance were applied to take into account clustering by practice.

**Model fitting and selection for patterns of ILI consultations 2010-2013**

To examine patterns of recording of ILI, we fitted negative binomial regression models with number of consultations as the outcome variable, and person-time at risk as the offset, to data from the last three seasons of the study period (2010/11, 2011/12 and 2012/13). We included age group, gender, winter season (here as a categorical variable) and Townsend quintiles as covariates. From preliminary analyses, we hypothesised that patterns of recording by age group would be different by gender; we therefore included a gender : age group interaction term.

We also used this model to examine practice-level variation in consultation rates, by predicting the number of ILI consultations per practice, based on age group, gender, winter season and Townsend quintile. We then divided the observed by the predicted number of consultations to calculate standardised consultation ratios (SCRs) by practice. We used funnel plots,[[2](#_ENREF_2)] with 95% control limits to compare the SCRs against an SCR of 1, which would be expected if observed and expected consultation rates were equal. We adjusted the 95% control limits for overdispersion by multiplying the variance by a constant term, derived using 10% Winsorisation.[[2](#_ENREF_2)]

**References**

1. Harrell FE. *Regression Modeling Strategies: With Applications to Linear Models, Logistic Regression, and Survival Analysis.* New York: Springer, 2001.

2. Spiegelhalter DJ. Funnel plots for comparing institutional performance. *Stat Med* 2005;**24**:1185-202.
